# EEG-informed fMRI analysis during a hand grip task: estimating the relationship between EEG rhythms and the BOLD signal

**DOI:** 10.3389/fnhum.2014.00186

**Published:** 2014-04-01

**Authors:** Roberta Sclocco, Maria G. Tana, Elisa Visani, Isabella Gilioli, Ferruccio Panzica, Silvana Franceschetti, Sergio Cerutti, Anna M. Bianchi

**Affiliations:** ^1^Department of Electronics, Information and Bioengineering, Politecnico di MilanoMilan, Italy; ^2^BIND - Behavioral Imaging and Neural Dynamics Center, University “G. d'Annunzio”Chieti, Italy; ^3^Department of Medicine and Aging Science, University “G. d'Annunzio”Chieti, Italy; ^4^Fondazione IRCCS Istituto Neurologico “C. Besta”Milan, Italy

**Keywords:** EEG-fMRI, motor task, BOLD, GLM, neural correlates, alpha rhythm, beta rhythm

## Abstract

In the last decade, an increasing interest has arisen in investigating the relationship between the electrophysiological and hemodynamic measurements of brain activity, such as EEG and (BOLD) fMRI. In particular, changes in BOLD have been shown to be associated with changes in the spectral profile of neural activity, rather than with absolute power. Concurrently, recent findings showed that different EEG rhythms are independently related to changes in the BOLD signal: therefore, it would be also important to distinguish between the contributions of the different EEG rhythms to BOLD fluctuations when modeling the relationship between the two signals. Here we propose a method to perform EEG-informed fMRI analysis where the changes in the spectral profile are modeled, and, at the same time, the distinction between rhythms is preserved. We compared our model with two other frequency-dependent regressors modeling using simultaneous EEG-fMRI data from healthy subjects performing a motor task. Our results showed that the proposed method better captures the correlations between BOLD signal and EEG rhythms modulations, identifying task-related, well localized activated volumes. Furthermore, we showed that including among the regressors also EEG rhythms not primarily involved in the task enhances the performance of the analysis, even when only correlations with BOLD signal and specific EEG rhythms are explored.

## Introduction

The complementary features of electroencephalography (EEG) and blood oxygen level-dependent functional magnetic resonance imaging (BOLD fMRI) constituted the basis for recent developments in the integration of these neuroimaging modalities (Liu et al., 1998, 2006; Dale et al., 2000; Babiloni et al., 2005; He and Liu, 2008). In particular, one of the most popular approaches combines EEG and fMRI measurements by using temporal- or frequency-specific information derived from EEG to obtain regressors of interest used in the common General Linear Model (GLM) framework; this multimodal strategy is usually referred to as EEG-informed fMRI analysis and it differs from the classical fMRI analysis in its unique ability to selectively localize the fMRI correlates to specific neuronal events or rhythms (He and Liu, 2008). In most of the literature about EEG-informed fMRI analysis, the EEG spectral power (either corresponding to the entire range of frequencies or to one or more specific bands) is simply used as a regressor to find correlations with the BOLD signal, without taking into account the problem of what is the best way to model the “transfer function” between the two signals (Logothetis et al., 2001; Laufs et al., 2003; Moosmann et al., 2003; Feige et al., 2005; Scheeringa et al., 2011). More recently, some attempts have been made in order to achieve a better understanding of the frequency-dependent neurovascular coupling. In this framework, Goense and Logothetis (2008) used simultaneous intra-cortical LFP-BOLD recordings and a multiple regression model, in which activity in many different frequency bands, covering the entire LFP range of frequencies, was employed to predict BOLD activity in alert behaving monkeys. The results showed that all of the LFP bands explained a significant part of the BOLD response. Kilner et al. (2005) observed that, from the fMRI standpoint, a proportionality exists between neuronal activation and the relative metabolic demands or rate of energy dissipation, in 1/s units. Simultaneously, from the perspective of EEG, activation gives rise to a shift in the spectral profile toward higher frequencies, also in 1/s units. The authors linked therefore these two observations through a dimensional analysis, proposing a “Heuristic” model, hereinafter named HEU. Such model states that BOLD activations are accompanied by an increase of the “average” frequency of the EEG neural activity, and it defines the average in the root mean square (RMS) sense. The HEU model was then tested by Rosa et al. (2010) on EEG-fMRI data recorded during a visual stimulation, and showed the ability to provide a better fit than the model proposed by Goense and Logothetis (2008), where no shift in EEG spectral profile toward high frequencies had been considered. However, although the “Heuristic” model importantly accounts for a different weighting of the power spectrum as a function of the frequency, it is not able to discriminate between different contributions from the single EEG bands in explaining BOLD variance. This can be limiting in some experiments, where it could be important to understand whether the specific rhythms, which are known to be involved in the phenomenon under investigation, contribute to explain BOLD variance independently or not. Moving in this direction, a recent work by Scheeringa et al. (2011) was conducted in order to understand if, during a visual attention task in humans, alpha and beta EEG power contribute to BOLD fluctuations independently or not. The findings of this latter study showed that different rhythms are independently related to changes in the BOLD signal. To achieve this result, the BOLD time series were modeled including separate regressors for each band in the design matrix, without accounting, though, for the “Heuristic” effect within each regressor (i.e., the possible shift in the spectral profile within the considered frequency band).

To bridge this gap, we intend to study how specific EEG rhythms selectively contribute to the BOLD fluctuations, taking into account the “Heuristic” model theory (Kilner et al., 2005). To this aim, we properly modified the design proposed by Rosa et al. (2010) by constructing a design matrix including different regressors for each EEG rhythm. One regressor per rhythm was obtained, in order to preserve the rhythms distinction and the possibility to study different frequency bands separately while taking into account the shift toward higher frequencies of the power spectrum. We applied this method to a human EEG-fMRI dataset, where a unimanual hand grip paradigm was employed on healthy subjects to elicit task-related activity in motor cortex. We chose a motor task to test our model, since the fluctuations of specific EEG rhythms during motor performance are well documented in literature. It is well known, in fact, that limbs movements are associated to desynchronization and synchronization (ERD/ERS) patterns on scalp EEG, involving alpha and beta rhythms (Pfurtscheller and Lopes da Silva, 1999). In order to study how the different movement-related EEG rhythms contribute to BOLD signal, we regressed fMRI data onto convolved time courses of features extracted from the power spectrum of each band of interest. We extracted five single-band features obtained by weighting power values as a function of frequency (which we called “Heuristic-Bands” or HEU-B model). We compared the performance of our model with the HEU model as introduced by Rosa et al. (2010), and with the approach described in Goense and Logothetis (2008), Scheeringa et al. (2011), where fMRI data are regressed onto convolved power time courses of the bands of interest, ignoring the “Heuristic” effect (from now on, this latter model will be referred to as “Frequency-Bands” or FB model). The evaluation of differences between models was first performed by constructing three different GLMs (one per type of regressors modeling), thus generating single model maps; then, regressors from different models were included in a same design matrix and a statistical parametric mapping (SPM) approach was used, in order to perform a quantitative comparison. The different models were evaluated comparing their results to those obtained investigating the main response to the task, through a stimulus-onset (SO) based analysis.

## Materials and methods

### Subjects and experimental protocol

**Figure 1:**
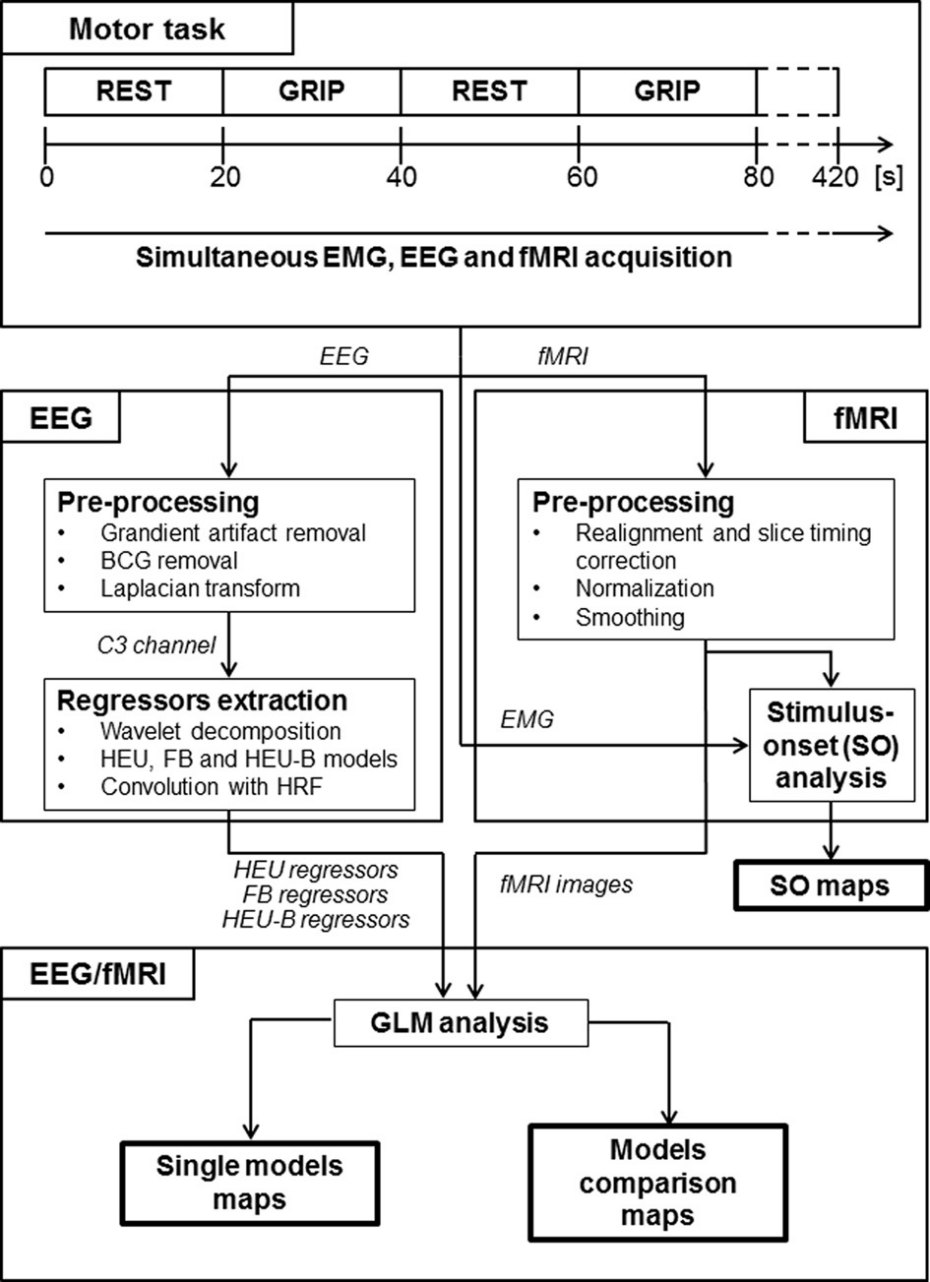
**Experimental protocol and data analysis pipeline.** This figure gives a schematic overview of the task, the different steps in EEG-fMRI processing and the outcome of the analysis (see Methods section for details). “Motor task” block: the motor task consists in a block-designed right hand grip. “EEG” block: gradient and BCG artifacts were removed from the EEG data; a surface Laplacian estimation was applied; EEG from channel C3 was decomposed using wavelet transform and HEU, FB and HEU-B regressors were estimated. “fMRI” block: the EPI images were motion and slice timing corrected, normalized and spatially smoothed; regressors modeling motor task were created and a stimulus-onset group map was obtained through GLM analysis. “EEG/fMRI” block: the HEU, FB, and HEU-B regressors were used to model EEG-fMRI relationships in separate design matrices and three single model maps (one per model) were obtained; finally, a direct comparison was performed by including regressors from different models in the same design matrix, thus obtaining model comparison maps.

Eleven (11) right handed healthy adult volunteers (7 male, 4 female, aged 35.6 ± 13.5 years) participated in the study performed at the “IRCCS Istituto Neurologico Carlo Besta”, Milan, Italy. All subjects had normal motor ability and no history of neurological or psychiatric disorders. The motor task consisted of 21 interleaved blocks of active and rest conditions. During the active condition the participants were instructed to squeeze a soft ball with the right hand, at 2 Hz rate, guided by a metronome. Blocks lasted for 20 s, resulting in an overall durations of 420 s (Figure 1). The switching instructions between the different conditions were given by video signals. All subjects were in a supine position with arms relaxed and head fixed with adjustable padded restraints on both sides. They were asked to move as little as possible throughout the experiment, to avoid blinking, and, in general, to keep their eyes open. All the subjects gave written informed consent to the experimental procedures that had gained Ethical Approval from the applicable institutional committees.

### EEG-fMRI acquisition

EEG was simultaneously acquired during fMRI scanning by using an MR-compatible EEG amplifier (SD MRI 32, Micromed, Treviso, Italy) and a cap providing 30 Ag/AgCl electrodes positioned according to the 10–20 system. An extra electrode was placed on the thorax to obtain an electrocardiogram (ECG). Concurrently, electromyographic activity was recorded by a pair of Ag/AgCl electrodes positioned 2–3 cm apart on either side of the right index flexor muscle. Electrodes impedances were kept below 5 kΩ. The signal was sampled at a rate of 1024 Hz using the software package provided by the manufacturer.

fMRI images were acquired on a 1.5 T MR scanner (Magnetom Avanto, Siemens AG, Erlangen, Germany). An axial gradient-echo echo-planar sequence was used to generate the functional images (*TR* = 2000 ms, *TE* = 50 ms, 21 slices, 2 × 2 mm^2^ in-plane voxel size, 4 mm slice thickness, no gap), resulting in a total of 210 functional scans for each subject. Whole-brain structural scans were also acquired using a T1-weighted sequence (160 slices, *TR* = 1640 ms, *TE* = 2 ms; 1 mm^3^ isotropic voxels), in order to obtain high-resolution anatomical images for each subject.

### EEG-fMRI data pre-processing

Gradient artifact, due to the EEG acquisition in an MRI environment, was removed off-line using the FMRIB plugin of the EEGLAB toolbox (http://www.fmrib.ox.ac.uk/eeglab/fmribplugin). The removal procedure was carried out by an initial subtraction of an average artifact template from each channel, as in (Allen et al., 2000), followed by an Optimal Basis Set (OBS) of principal components for the removal of artifact residuals (Niazy et al., 2005). The ballistocardiogram (BCG) artifact was also removed using the FMRIB plugin implementing OBS. Finally, a surface Laplacian estimation was applied to EEG data, in order to free them from a reference and make them spatially sharpened (Hjorth, 1975; Visani et al., 2011). Detailed results of EEG artifact removal procedure are shown in the Supplementary Materials section.

The fMRI images were motion and slice timing corrected, normalized to a standard EPI template based on neuroanatomical atlas of Talairach and Tournoux (1988). Finally, normalized images were spatially smoothed with an 8 × 8 × 8 mm full width at half maximum Gaussian kernel. All steps of fMRI data pre-processing were performed using the SPM5 software package (http://www.fil.ion.ucl.ac.uk/).

### EEG time-frequency analysis

In order to estimate the amplitude of neuroelectrical oscillatory activity, we decomposed the EEG signal into time-frequency domain. As in (Laufs et al., 2003; Moosmann et al., 2003; Horovitz et al., 2008; Ritter et al., 2009), we selected only the channel expected to be the most involved one in modulating oscillatory activity during the task. In our case, since the experimental protocol corresponds to a motor task, the central electrode on the contralateral motor area was chosen (C3), which is already known to be placed in the Rolandic area (Pfurtscheller and Lopes da Silva, 1999; Visani et al., 2006).

The signal extracted from C3 channels, $\tilde{s}(t)$, was analyzed in the time-frequency domain by convolution with complex Morlet wavelets, *w(f,t)*, having a frequency range from 1 to 40 Hz in 0.5 Hz steps. As in our previous work (Sclocco et al., 2012), the EEG bandwidth was limited to 40 Hz (low-gamma rhythm), since above that frequency MRI-related artifact are more difficult to remove, therefore preventing from obtaining a signal of good quality. The time-varying power of the signal around frequency *f* was then obtained by the squared modulus of the convolution (Tallon-Baudry and Bertrand, 1999): $P\text{​}\left(f,t\right)=\;{\left|w(f,t)*\tilde{s}(t)\right|}^{2}$ (Figure 1).

### Modeling EEG-fMRI relationship

After the power spectrum for all frequencies and time points, *P(f,t)*, was obtained, we extracted regressors for subsequent EEG-informed fMRI analysis.

Starting from the time-frequency decomposition of the EEG signal, we obtained fMRI regressors using three models of transfer function between EEG and fMRI (Figure 1).

The first model, HEU, assumes that the increasing BOLD signal is associated with a shift in the EEG spectral profile toward higher frequencies, as in (Kilner et al., 2005; Rosa et al., 2010). The equation describing the regressor is directly derived from the dimensional analysis by Kilner et al. (2005). Briefly, the authors measure the effect of activation on the EEG signal through the roughness of the signal, defined as the normalized variance of the first temporal derivative of the EEG. Since the roughness is mathematically equivalent to the negative curvature of the EEG autocorrelation function evaluated at zero lag, the Wiener-Khinchin theorem allows to express the relationship in terms of spectral density, and consequently the activation in terms of the “normalized” spectral density. The regressors are obtained by the integration, over the whole EEG spectral range, of the time-frequency power values multiplied by the square of the corresponding frequency. HEU regressors are thus defined as follows:

$$r_{HEU}\text{​}\left(t\right)=\sqrt{\sum_{f=1}^{n_{f}}f^{2}\tilde{P}(f,t)},$$

where $\tilde{P}$ is the normalized power spectrum and *n*_*f*_ is the number of frequencies considered in the time-frequency decomposition.

For the other two models, we created one regressor for each of the five EEG canonical rhythms [δ (1–4 Hz), θ (5–8 Hz), α (8–12 Hz), β (12–30 Hz), and γ (30–40 Hz)]. Although the EEG frequency content of interest during motor performance is known to be mainly in alpha and beta bands (i.e., the so-called “Rolandic rhythms”), we chose to include in the fMRI design matrix a set of regressors covering the entire range of the available EEG frequency bandwidth, following the findings from recent studies (Goense and Logothetis, 2008).

The second model, FB, considers the aforementioned five bands of neural activity and extracts a regressor from each of them by integrating the values of the time-frequency power spectrum over the corresponding frequency range, *a* = [*f*_*min*_, *f*_*max*_]:

$$r_{FB}\text{​}{\left(t\right)}_{a}=\sum_{f\;=\;f_{\text{min}}}^{f_{max}}P\text{​}\left(f,\;t\right).$$

The third model, HEU-B, considers the same bands of FB model, but defines each band regressor accounting for the “Heuristic” effect. The five regressors of the HEU-B model are, therefore, given by:

$$r_{HEU\;-\;B}{\left(t\right)}_{a}=\sqrt{\sum_{f\;=\;f_{\text{min}}}^{f_{\text{max}}}f^{2}\tilde{P}\text{​}\left(f,\;t\right),}$$

where $\tilde{P}$ indicates the normalized power spectrum and *a* = [*f*_*min*_, *f*_*max*_] is the bandwidth of the considered EEG rhythm.

HEU-B model allows, at the same time, to model the dependence of BOLD response on modulations of the EEG spectral profile and to separate the response to different frequency bands, making possible to identify the BOLD correlates to a specific EEG rhythm (e.g., Rolandic rhythms).

### Stimulus onset-based fMRI analysis

In order to investigate the effect of the experimental task, a SO fMRI analysis was first performed using regressors modeling the alternation of active and rest blocks. The onsets of active blocks were detected from the EMG recording. The bursts of activity corresponding to the grip of the right hand were identified by a physiologist and a SO box-car function synchronous with EMG bursts was created. At a first stage of analysis, the fMRI data of each participant were analyzed using the mass univariate approach based on GLM theory, as implemented in SPM5. The expected response was modeled as the convolution of the SO box-car function with the SPM canonical Haemodynamic Response Function (HRF), including its multivariate first order Taylor expansion in time (time derivative) and width (dispersion derivative). Movement parameter estimates produced by realignment procedure were also included as confounding regressors, in order to remove residual movement artifacts (Friston et al., 1996).

The whole set of regressors modeling the effects of interest and the unwanted effects forming the first level design matrix is then fitted to the image data of each subject involved in the experiment. After the estimation of the regression coefficients, inference on relevant contrasts of their estimates was performed using a Student's *t* statistic. A first-level *t*-contrast was specified for each basis function, resulting in 3 *t*-contrast maps for participant. At a second stage of analysis, individual contrast maps were then included into a second-level full factorial design, in order to perform an RFX (random-effects) analysis. Within-subject One-Way analysis of variance (ANOVA) was computed, and inference was carried out using an *F*-test.

We then performed anatomical and functional labeling of the involved areas using the probability maps from Anatomical Automatic Labeling (AAL) SPM toolbox (http://www.cyceron.fr/web/aal_anatomical_automatic_labeling.html) and SPM Anatomy Toolbox (Eickhoff et al., 2005).

### EEG-informed fMRI analysis

#### Single-model mapping

In order to investigate the link between neuroelectrical activity and BOLD signal, further fMRI analyses were performed using EEG-derived regressors. At first-level analysis, three design matrices were obtained for each subject, using different groups of regressors *r*_*HEU*_(*t*), *r*_*FB*_(*t*)_*a*_, and *r*_*HB*_(*t*)_*a*_.

*r*_*HEU*_(*t*), *r*_*FB*_(*t*)_*a*_, and *r*_*HB*_(*t*)_*a*_ time series were downsampled to match the SPM canonical HRF sampling rate, which we set to slice acquisition time interval (TR/number of slices) (Josephs et al., 1997; Henson and Friston, 2007). The time series were then convolved with the canonical HRF and with its time and dispersion derivatives. The results of the convolution were used to construct three individual GLMs (one for each model) that were then fitted to the fMRI data. As in the SO analysis, the parameters obtained from motion correction during images pre-processing were also included in the GLM. Inference on the estimated regressors was performed using *t*-tests. A first-level *t*-contrast was specified for each basis function of the *r*_*HEU*_(*t*) regressors, as well as for alpha and beta regressors of *r*_*FB*_(*t*)_*a*_ and *r*_*HB*_(*t*)_*a*_ models. Although all the five EEG rhythms were included in the first level design matrices, for the sake of simplicity, inference was performed only on the alpha and beta frequency rhythms. This approach was adopted since it is known that the modulation of the motor-related oscillatory activity is mainly focused in the alpha and beta frequency ranges (Papakostopoulos et al., 1980; Pfurtscheller, 1981; Salmelin and Hari, 1994; Salmelin et al., 1995; Visani et al., 2006). Further analyses investigating other rhythms could be an interesting extension of the proposed approach, but this goes beyond the scope of the present work. The outcome of the first level analysis, consisted in 15 *t*-contrast maps for each subject (3 maps for HEU, 6 for FB and HEU-B). An example of final regressors from each model for a representative subject is shown in Figure 3.

At the second stage of analysis, 5 full factorial designs were implemented (HEU, FB-alpha, FB-beta, HEU-B-alpha, HEU-B-beta), each design including 3 individual contrast maps per participant. A total of 5 within-subject One-Way analysis of variance (ANOVA) was computed and inferences were carried out through an *F*-test.

In order to test whether the choice to include all of the five EEG rhythms in the first-level designs would help to explain the relationship with the BOLD signal, or it would be enough to consider alpha and beta rhythms only, we built two further first-level design matrices for each subject (one for FB and one for HEU-B), including alpha and beta regressors only. 12 further first-level *t*-contrast maps for participant (6 for each model) were obtained, and a second-level analysis was performed similarly to the one described above. We called the resulting images “reduced models comparison” maps.

#### Models comparison mapping

In order to compare the different models and to understand which one is best suited for investigating the BOLD response to EEG frequency oscillations in the alpha and beta bands during a motor task, at the first-level of the analysis, we included all the regressors obtained from FB and HEU-B models in the same design matrix. Given the poor performance of HEU model in single-model mapping (see Results section below), it was not included in this further step of the analysis. The use of a single GLM design matrix comprising different models allowed to highlight BOLD variability that could be explained by a specific model and not by others (Friston et al., 1994; Rosa et al., 2010). As for the single model mapping, first-level t-contrasts were specified only for alpha and beta regressors of the *r*_*FB*_(*t*)_*a*_ and *r*_*HB*_(*t*)_*a*_ models. This resulted in a total of 12 first-level *t*-contrast maps for participant (6 for FB and 6 for HEU-B). A second-level analysis was thus implemented in order to compare the performance of FB and HEU-B model.

At a group level, 4 full factorial designs were then implemented, each design including 3 *t*-contrast maps per subject. Within-subject One-Way analysis of variance (ANOVA) inferences on the estimated regressors were performed using *F*-tests and 4 final “global models comparison” maps were obtained. This analysis approach allowed us to explore the neural correlates of EEG regressors that are uniquely attributable to each model within the pair.

#### Labeling of the active areas

For each “single-model” and for each “models comparison” map, we performed anatomical labeling of the resulting areas. In order to evaluate the ability of the different models to identify the task-related activations, we generated a “BOLD activation mask” (BAM) including the functional areas resulting from SO analysis (Rosa et al., 2010). Accordingly, we built two main classes of brain areas: “motor” areas, corresponding to BAM functional regions, and “non-motor” areas, otherwise. The performances of the different models were thus assessed by evaluating the F-scores, the number of voxels above the adopted *p*-value thresholds, as well as the location of these voxels inside and outside the BAM (Rosa et al., 2010).

## Results

### Stimulus onset-based fMRI analysis

**Figure 2:**
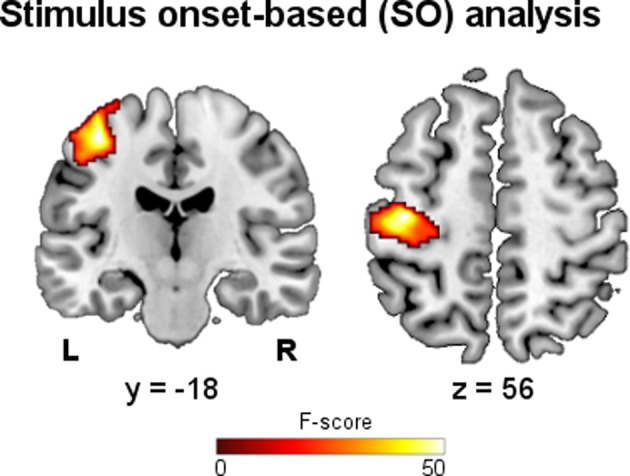
**Stimulus onset-based analysis.** Group map showing the main effect of right hand grip on fMRI data (*p* < 0.05, FWE).

The results of the SO fMRI analysis showed activations related to the performed motor task. The main effect of hand grip resulting from the group analysis is shown in Figure 2, while in Table 1 the significant clusters are assigned to anatomical and functional regions. As expected, SO-related activations were found in functional areas that are well known to be involved in motor execution: left primary motor cortex (M1-BA4), premotor cortex (PM-BA6), primary somatosensory cortex (S1-BAs 1, 2 and 3), and supplementary motor area (SMA-BA6).

**Table 1:** **Stimulus onset-based fMRI analysis**.

**Cluster size (# voxel)**	**Maximum MNI coordinates (mm)**	**Maximum F-score (*p* < 0.05, FWE^a^)**	**Anatomical areas**	**Brodmann areas (BA) (# voxels assigned)**
914	−40, −22, 61	51.29	54% Left pre-central gyrus, 45% left pre-central gyrus	BA6 (386), BA4a (135), BA1 (104), BA4p (103), BA3b (93), BA2 (25), BA3a (15)

a *Family wise error corrected for multiple comparison*.

### EEG-informed fMRI analysis

**Figure 3:**
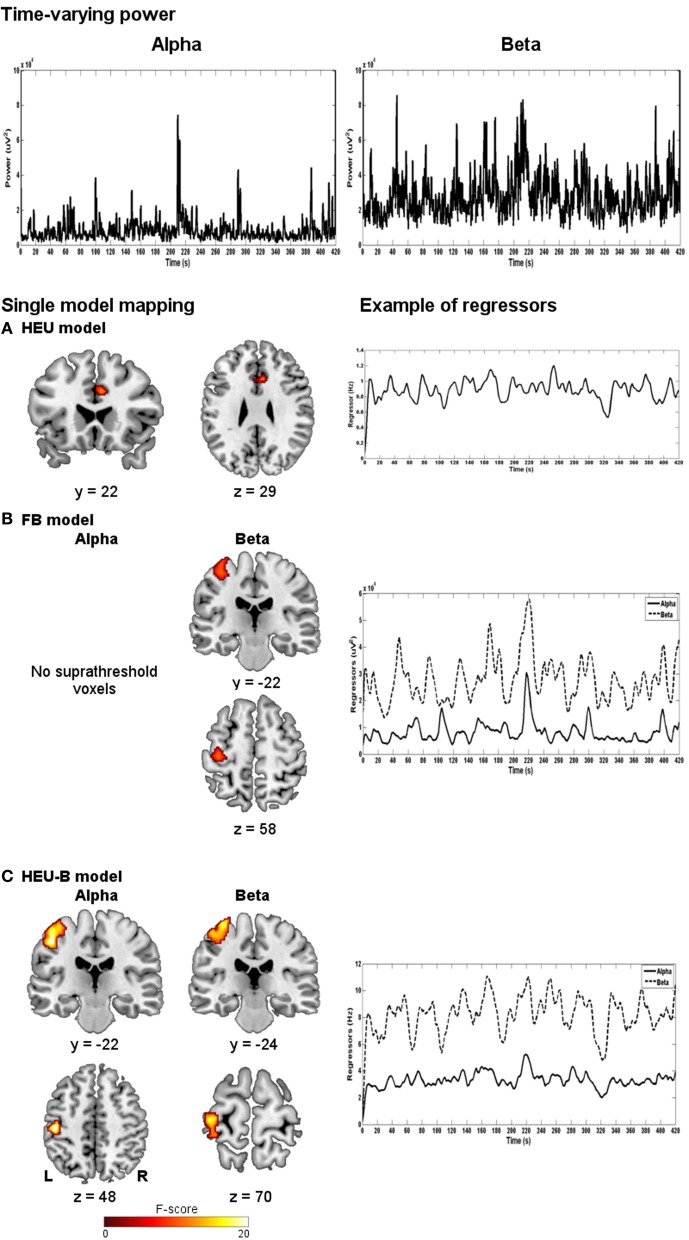
**Single-model mapping.** Top panel: alpha and beta time-varying EEG power for a representative subject. Bottom panel: group maps obtained using HEU **(A)**, FB **(B)** and HEU-B **(C)** models, along with examples of regressors for a representative subject (after downsampling and convolution with HRF). For FB and HEU-B models, neural correlates for alpha and beta rhythms are shown (HEU: *p* < 0.001, uncorrected; FB: *p* < 0.001, uncorrected; HEU-B: *p* < 0.05, FDR).

In the following analyses, we explored the relationship between EEG and BOLD for each model described in the Methods section. Figure 3 shows the neural correlates of the EEG regressors obtained using the HEU model (A), the FB model (B) and the HEU-B model (C). For the two latter models, maps relative to contrasts on alpha and beta rhythms are shown. In Table 2, the significant clusters are listed and assigned to anatomical and functional regions for each model and each contrast.

**Table 2:** **Single model mapping**.

**HEU MODEL**					
	**Cluster size (# voxel)**	**Maximum MNI coordinates (mm)**	**Maximum F-score (*p* < 0.001, uncorrected)**	**Anatomical areas**	**Brodmann areas (BA) (# voxels assigned)**
	75	6, 16, 35	13.82	60% Right middle cingulate cortex, 40% left middle cingulate cortex	BA24 (75)
**FB MODEL**					
	**Cluster size (# voxel)**	**Maximum MNI coordinates (mm)**	**Maximum F-score (*p* < 0.001, uncorrected)**	**Anatomical areas**	**Brodmann areas (BA) (# voxels assigned)**
**Alpha**	–	–	–	–	–
**Beta**	207	−38, −26, 63	11.11	86% Left pre-central gyrus, 14% left post-central gyrus	BA6 (126), BA4a (56), BA4p (13), BA3b (6), BA1 (1)
**HEU-B MODEL**					
	**Cluster size (# voxel)**	**Maximum MNI coordinates (mm)**	**Maximum F-score (*p* < 0.05 FDR^a^)**	**Anatomical areas**	**Brodmann areas (BA) (# voxels assigned)**
**Alpha**	585	−48, −26, 53	25.35	65% Left post-central gyrus, 34% left pre-central gyrus	BA6 (156), BA41 (121), BA1 (114), BA3b (88), BA2 (72), BA4p (10)
**Beta**	473	−34, −28, 75	17.25	56% Left pre-central gyrus, 42% left post-central gyrus	BA6 (210), BA4a (93), BA1 (80), BA3b (35), BA4p (14), BA2 (3), BA3a (1)

a False Discovery Rate corrected for multiple comparison.

**Figure 4:**
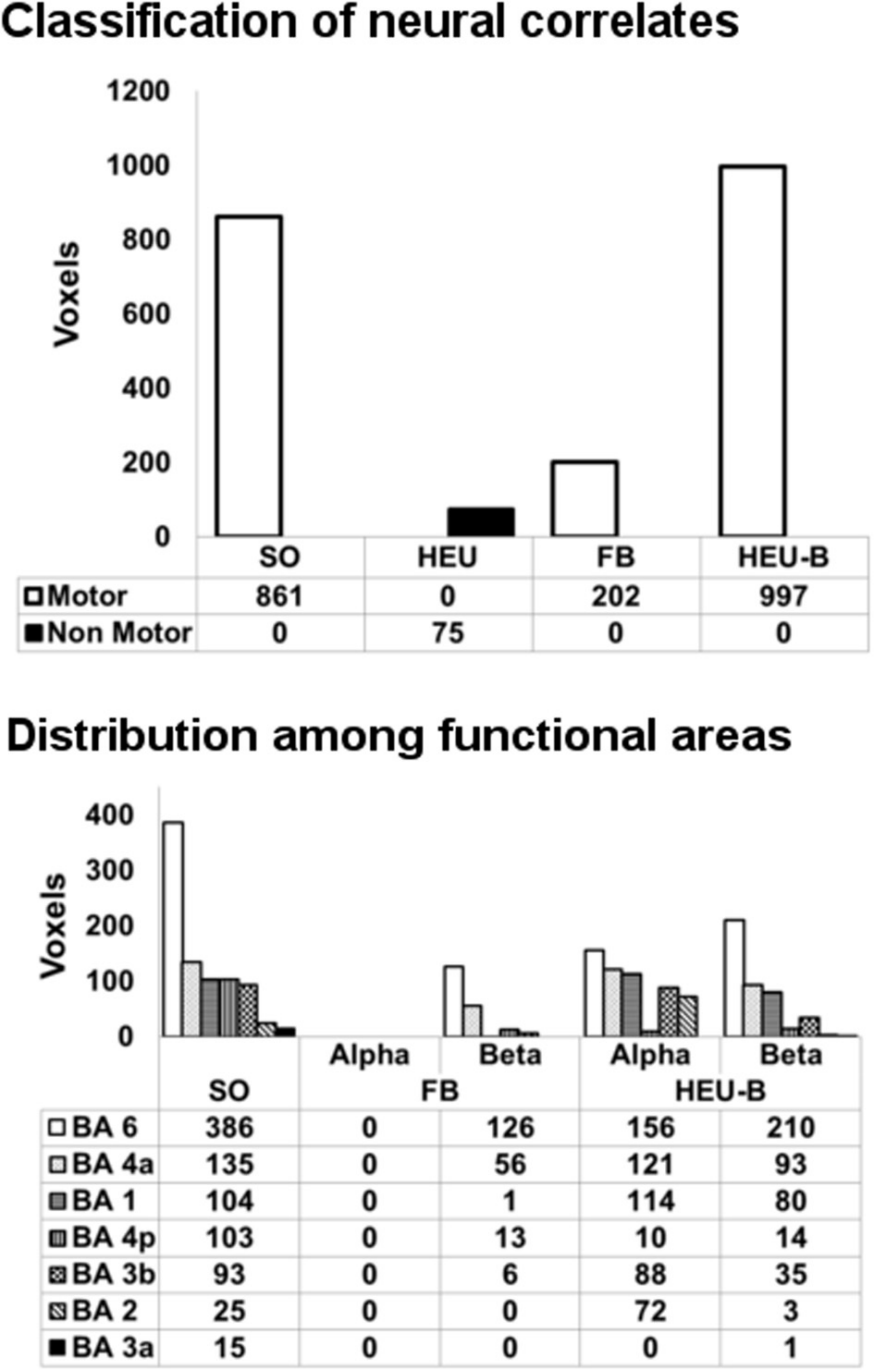
**Classification of neural correlates from single model mapping.** Top panel: voxels classification according to the BAM (voxels belonging to the same areas activated in SO analysis are classified as “motor” voxels). Bottom panel: distribution of “motor” voxels among the Brodmann areas identified by the SO analysis. Since HEU model only produced “non motor” voxels, it was not included in this second classification. The number of voxels assigned to the classes are tabulated below bars.

In Figure 4 (top panel), the voxels of the active areas are classified as “motor” and “non-motor”, according to the previously defined BAM. The “motor” voxels were further classified according to their Brodmann areas (Figure 4, bottom panel).

The HEU model produced only voxels outside the BAM, in particular located in the ventral portion of middle cingulate cortex (MCC) (Figure 3A); therefore, it was excluded from the second classification based on Brodmann areas, and from the following model comparison mapping analyses.

The FB model revealed one active cluster only relatively to the beta rhythm (Figures 3B, 4; Table 2). This cluster, entirely included in the BAM, is mostly located in M1 (BA 4) and PM (BA 6).

The HEU-B model produced the highest number of “motor” voxels (997), and no voxels outside the BAM were found. Furthermore, voxels distribution among the motor areas are very similar to those resulting from the SO fMRI analysis, since both alpha and beta related activations were located in S1 (BAs 1, 2 and 3), M1 (BA 4), SMA (BA6), and PM (BA 6) (Figures 3C, 4; Table 2), thus revealing a co-localization of the neural correlates of alpha and beta rhythms with task-related BOLD activity.

**Figure 5:**
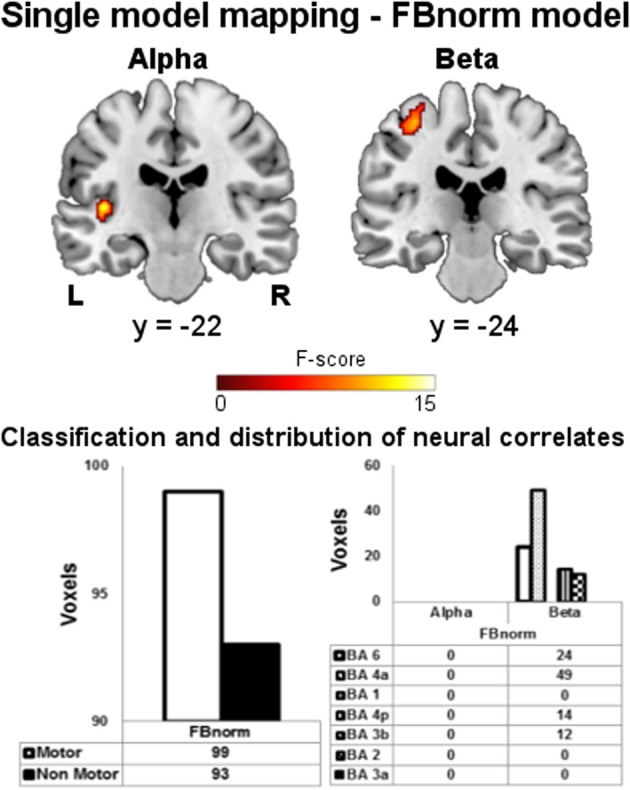
**Single model mapping—FBnorm model.** Top panel: group maps obtained from single model mapping using FBnorm model. Neural correlates for alpha and beta rhythms are shown (*p* < 0.001, uncorrected). Bottom panel: (left) voxels classification according to the BAM; (right) distribution of “motor” voxels among the Brodmann areas identified by the SO analysis. The number of voxels assigned to the classes are tabulated below bars.

Before performing the following pairwise comparison between FB and HEU-B models, we also investigated whether the FB model performance could be increased by using the normalized spectral power instead of the absolute one. Therefore, a further single-model mapping analysis was performed (we called the model FBnorm), the results of which are shown in Figure 5. The FBnorm model was able to provide BOLD correlates also for alpha rhythm, but producing only “non-motor” voxels. The results relative to the beta rhythm are similar to the FB model (Figures 3B, 4) since only one cluster was found being entirely within the BAM and mostly located in M1 (BA 4) and PM (BA 6). However, the number of resulting voxels is decreased if compared with FB model results (FBnorm: 99 voxels; FB: 202 voxels).

**Figure 6:**
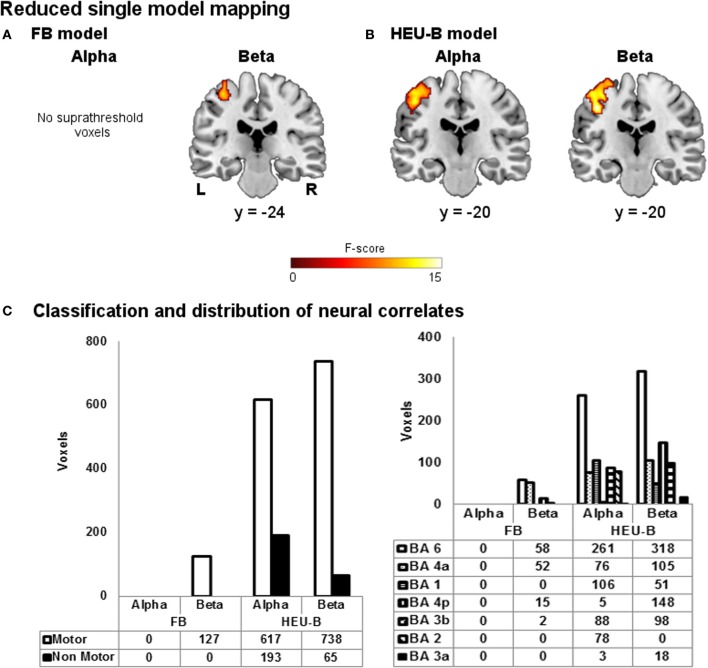
**Reduced single model mapping.** Group maps obtained showing neural correlates of alpha and beta rhythms for **(A)** reduced FB model and **(B)** reduced HEU-B model (FB: *p* < 0.001, uncorrected; HEU-B on alpha: *p* < 0.001, uncorrected; HEU-B on beta: *p* < 0.05, FDR). **(C)** Voxels classification according to the BAM (left); distribution of “motor” voxels among the Brodmann areas identified by the SO analysis (right). The number of voxels assigned to the classes are tabulated below bars.

**Figure 7:**
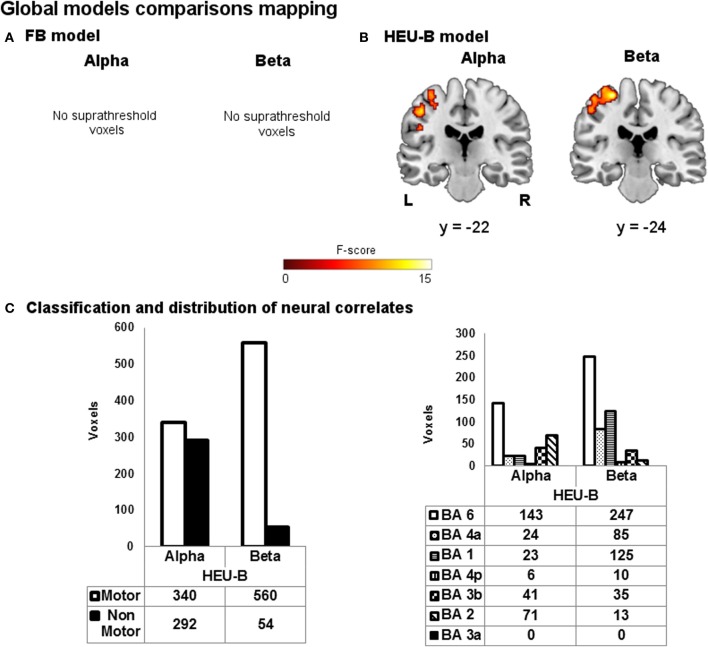
**Global models comparison mapping.** Group maps showing neural correlates of alpha and beta rhythms for **(A)** FB and **(B)** HEU-B model (*p* < 0.001, uncorrected). **(C)** Voxels classification according to the BAM (left); distribution of “motor” voxels among the Brodmann areas identified by the SO analysis (right). The number of voxels assigned to the classes are tabulated below bars.

The reduced single model maps are shown in Figure 6. As it can be noticed, the ability of the models to capture the correlation with task-induced BOLD variations turned out to be decreased. In particular, the BOLD correlates of beta rhythm relative to the reduced FB model identified less “motor” voxels than the FB one (reduced FB: 127 voxels; FB: 202 voxels), while the reduced HEU-B model produced voxels outside the BAM when contrasting on both alpha (193 voxels) and beta (65 voxels) regressors (see Figures 4, 6C). Therefore, including among the regressors also the EEG rhythms not primarily involved in motor activity enhances the performance of the EEG-informed fMRI analysis, allowing a better explanation of BOLD signal variation. The results of the model comparison mapping between Frequency Bands and Heuristic Bands models are shown in Figure 7 and Table 3. As it was found in the previously described analysis, the HEU-B model was the only one that produces active areas within the motor regions, especially with reference to the beta rhythm. The HEU-B model in the beta frequency range produced a higher number of “motor” voxels (550) and a lower number of “non-motor” voxels than in the alpha frequency range. Alpha related activations were located mainly in SMA (BA6), S1 (BAs 2,3), whereas beta related active clusters were distributed mainly between SMA (BA6), S1 (BA1), and M1 (BA4) (Figure 7C, Table 3). Differently from HEU-B model, no voxel survived the uncorrected threshold in the FB model, for both the alpha and beta rhythms.

**Table 3:** **Global models comparison**.

**FB MODEL**					
	**Cluster size (# voxel)**	**Maximum MNI coordinates (mm)**	**Maximum F-score (*p* < 0.001, uncorrected)**	**Anatomical areas**	**Brodmann areas (BA) (# voxels assigned)**
**Alpha**	–	–	–	–	–
**Beta**	–	–	–	–	–
**HEU-B MODEL**					
	**Cluster size (# voxel)**	**Maximum MNI coordinates (mm)**	**Maximum F-score (alpha: p<0.001, uncorrected; beta: p<0.05, FDR)**	**Anatomical areas**	**Brodmann areas (BA) (# voxels assigned)**
**Alpha**	243	−46, −22, 48	11.58	42% Left post-central gyrus, 41% left pre-central gyrus, 16% left inferior parietal lobule	BA6 (75), BA2 (71), BA3b (41), BA4a (24), BA1 (23), BA4p (6)
	97	−24, −6, 66	13.24	63% Left superior pre-frontal gyrus, 29% left pre-central gyrus	BA6 (68)
	79	52, −64, −4	15.78	100% Right middle temporal gyrus	BA19 (79)
	76	−46, −26, 30	10.15	39% Left supramarginal gyrus, 22% left post-central gyrus, 13% left inferior parietal lobule	BA7 (31), BA40 (18)
	71	−38, −36, −4	14.32	11% Left hippocampus, 5% left inferior temporal gyrus	BA27 (8), BA20 (4)
**Beta**	560	−28, −24, 64	15.25	55% Left pre-central gyrus, 42% left post-central gyrus	BA6 (247), BA1 (125), BA4a (85), BA3b (35), BA2 (13), BA4p (10)
	54	−4, 26, 36	10.51	78% Left middle cingulate cortex, 20% left superior frontal cortex	BA24 (41), BA9 (11)

## Discussion

In this paper, we investigated the relationship between neural activity and BOLD signal in simultaneously acquired EEG and fMRI data during a motor task in healthy human subjects. We compared three different models (Heuristic, Frequency Bands and Heuristic Bands) used to build regressors from the EEG signal, in order to explore the correlations between each of them and the fMRI data. For the evaluation of the performances of the different models, we chose to use data acquired during a motor task, where functional activations related to the task execution are well known.

The present work showed that the correlations between BOLD signal and EEG power fluctuations are better captured by models which take into account the different EEG rhythms, relatively to the identification of the motor-related activations. In fact, both the Heuristic Bands model and, even if to lesser extent, the Frequency Bands model, showed correlations consistent with the motor task. On the contrary, HEU model only produced voxels not belonging to the functional areas involved in the motor task. Considering the differences between the EEG rhythms by modeling them with separate GLM regressors, though, didn't turn out to be enough for a good performance of the model. Indeed, the Frequency Bands model wasn't able to capture as much of the BOLD signal variance as the Heuristic Bands one. It was therefore important to take into account also the EEG spectral shift toward higher frequencies during activation, as predicted by Kilner et al. (2005) and tested in Rosa et al. (2010). In particular, at the best of our knowledge, our results showed for the first time that considering the EEG spectral shift separately for each single rhythm improves the ability of the model to capture the variance of the BOLD signal. Using EEG regressors estimated by HEU-B model in the GLM framework improved the statistical significance of the results (HEU-B *F*-maps are FDR-corrected for multiple comparisons, while no voxel survived a correction for multiple comparisons in FB-related maps). This result is consistent with the findings of our pilot study (Sclocco et al., 2012), where the HEU and HEU-B models were compared in a similar way as in this work. Here, we provide a stronger support to previous results, using a larger dataset which also allowed the random-effects approach in the group analyses.

Moreover, we investigated the influence of the power normalization on the FB model, in order to verify whether using normalized power would improve its performance. Results were very similar to those obtained with the un-normalized FB model, providing additional support to the importance of considering the “Heuristic” effect to model the relationship between EEG and BOLD signals.

As for the areas identified by the HEU-B model, most of them are consistent with the broad literature available on the subject. Electrocorticographic (Gastaut, 1952; Papakostopoulos et al., 1980) and neuromagnetic (Salmelin and Hari, 1994) recordings have shown that the Rolandic beta rhythm mainly originates in the anterior bank of the central sulcus, while the Rolandic alpha rhythm concentrates predominantly in the post-central cortex. However, other studies reported rather widespread and individually variable cortical desynchronization during movement in both the pre-central and post-central cortex (Crone et al., 1998b). Also in magnetoencephalographic (Willemse et al., 2010) and EEG-fMRI (Parkes et al., 2006) studies, both the Rolandic alpha and beta rhythms are found in pre- and post-central areas. Thus, the association of the Rolandic alpha rhythm with somatosensory cortex and the Rolandic beta with motor cortex is not definitive. Our study contributes in this sense by showing a predominant location of alpha-related cluster in the post-central areas (65% of the cluster), while the beta-related area mainly involved the anterior portion of the central sulcus (56% of the cluster) (Table 2). Furthermore, the distribution of resulting neural correlates among the functional motor areas revealed some differences between the alpha and beta localizations. Indeed, contrast on alpha regressor produced more voxels corresponding to the primary somatosensory cortex (alpha: 274 voxels; beta: 119 voxels); on the other hand, most of the beta-related voxels were found in the primary motor cortex and pre-motor cortex, respectively (alpha: 287 voxels; beta: 317 voxels).

Finally, in the pairwise comparison performed between FB and HEU-B models (Figure 7, Table 3), the contrast vector analysis on the alpha regressor in the HEU-B model identified other functional areas in addition to task-related correlates: in particular, correlations are found in BAs 7, 19, and 40. Although the presence of these extra-rolandic clusters could be due to the uncorrected threshold adopted for the alpha contrast, they can be interpreted in the context of an attentional network active during the rest blocks (Ritter et al., 2009). At this regard, previous studies showed how BA 7 (i.e., the somatosensory association cortex) is active during the expectation of an event (MacKay and Crammond, 1987). Also the involvement of BA 40 and BA 19 (i.e., supramarginal gyrus and middle temporal area, respectively) can be explained in the context of the attentional network (Cooreman et al., 2011): left supramarginal gyrus has been shown to be important for attention in relation to limb movements (Rushworth et al., 1997, 2001), while middle temporal area contains the MT/V5 area is an associative visual region associated with attentional modulation (Büchel and Friston, 1997; Coull, 1998).

The present study has a main limitation that should be considered, that is, the choice of a single EEG electrode (C3), without taking into account the information carried by other channels. Still, the aim of this work is the comparison of EEG-to-BOLD transfer functions within a motor task, therefore a certain degree of simplification is needed in order to identify the primary sources of information related to the investigated protocol. Future developments of this study could focus on the neural correlates of other recording sites. The relationship between alpha rhythm and BOLD signal, for example, has been extensively investigated during the last decade, especially during task-free “resting-state” studies; spontaneous fluctuations in alpha oscillation power has been noted to negatively correlate with activity in the dorsal attention system of superior frontal and intraparietal regions (Laufs et al., 2003; Mantini et al., 2007), while a positive correlation was found with activity in a cingulo-opercular network encompassing dorsal anterior cingulate cortex, frontal operculum/anterior insula and thalamus (Dosenbach et al., 2007; Sadaghiani et al., 2010). More recently, phase synchronization of alpha oscillations across distant cortical regions have been used in order to confirm the existence of a link between the coupling in the upper alpha band and the fronto-parietal network (Sadaghiani et al., 2012), suggested to be responsible for the phasic control of alertness and task requirements, hence complementing previous findings relating alpha oscillation power to neural structure serving tonic control (Sadaghiani et al., 2010). Interestingly, the authors were able to relate different electrophysiological signatures (power vs. phase locking, positive vs. negative correlation) to distinct functional networks involved in cognitive control and spatial attention. Therefore, using the proposed HEU-B model in order to explore the neural correlates of frontal or parietal electrodes could give new insights on the relationship between specific EEG rhythms and attention-related functional connectivity networks during an active task. Further extensions of the present work could be also studying the BOLD correlates of the EEG rhythms other than alpha and beta, in order to investigate their involvement in the motor task. For example, desynchronization patterns, restricted to the onset of movement, are reported in the literature in the high gamma frequency range (60–100 Hz) (Crone et al., 1998a; Marsden et al., 2000; Ohara et al., 2001), as well as increased theta activity preceding the movement onset (Popivanov et al., 1999; Turak et al., 2001).

However, the purpose of the present study is the identification of the best way to model the relationship between EEG and BOLD signals, while the complete description of the whole-brain neural correlates found during a motor task could be referred to a further investigation. Understanding the nature of the link between neuronal activity and BOLD signal plays a crucial role in improving the interpretability of BOLD imaging and relating electrical and hemodynamic measures of human brain function. Finding the optimal way to model the relationship between these two techniques is nowadays an open issue. We expect this work to be a starting point for studying other types of EEG rhythms, and other types of task-related activations/deactivations.

## Author contributions

All the authors substantially contributed to the conception and design of the work, as well as the acquisition, analysis and interpretation of data. Furthermore, the work was drafted, critically revised and its final version was approved by all of the authors.

## Conflict of interest statement

The authors declare that the research was conducted in the absence of any commercial or financial relationships that could be construed as a potential conflict of interest.

## References

[B1] Allen P. J. Josephs O. Turner R. (2000). A method for removing imaging artifact from continuous EEG recorded during functional MRI. Neuroimage 12, 230–239 10.1006/nimg.2000.0599 10913328

[B2] Babiloni F. Cincotti F. Babiloni C. Carducci F. Mattia D. Astolfi L. (2005). Estimation of the cortical functional connectivity with the multimodal integration of high-resolution EEG and fMRI data by directed transfer function. Neuroimage 24, 118–131 10.1016/j.neuroimage.2004.09.036 15588603

[B3] Büchel C. Friston K. (1997). Modulation of connectivity in visual pathways by attention: cortical interactions evaluated with structural equation modelling and fMRI. Cereb. Cortex 7, 768–778 10.1093/cercor/7.8.768 9408041

[B4] Cooreman C. Sclocco R. Tana M. G. Vanderperren K. Visani E. Panzica F. (2011). BOLD correlates of Alpha and Beta EEG-rhythm during a motor task, in Neural Engineering (NER), 2011 5th International IEEE/EMBS Conference on (Cancun), 25–28 10.1109/NER.2011.591048118465747

[B5] Coull J. T. (1998). Neural correlates of attention and arousal: insights from electrophysiology, functional neuroimaging and psychopharmacology. Prog. Neurobiol. 55, 343–361 10.1016/S0301-0082(98)00011-2 9654384

[B6] Crone N. E. Miglioretti D. L. Gordon B. Lesser R. P. (1998a). Functional mapping of human sensorimotor cortex with electrocorticographic spectral analysis. II. Event-related synchronization in the gamma band. Brain 121, 2301–2315 10.1093/brain/121.12.2301 9874481

[B7] Crone N. E. Miglioretti D. L. Gordon B. Sieracki J. M. Wilson M. T. Uematsu S. (1998b). Functional mapping of human sensorimotor cortex with electrocorticographic spectral analysis. I. Alpha and beta event-related desynchronization. Brain 121, 2271–2299 10.1093/brain/121.12.2271 9874480

[B8] Dale A. M. Liu A. K. Fischl B. R. Buckner R. L. Belliveau J. W. Lewine J. D. (2000). Dynamic statistical parametric mapping: combining fMRI and MEG for high-resolution imaging of cortical activity. Neuron 26, 55–67 10.1016/S0896-6273(00)81138-1 10798392

[B9] Dosenbach N. U. Fair D. A. Miezin F. M. Cohen A. L. Wenger K. K. Dosenbach R. A. (2007). Distinct brain networks for adaptive and stable task control in humans. Proc. Natl. Acad. Sci. U.S.A. 104, 11073–11078 10.1073/pnas.0704320104 17576922 PMC1904171

[B10] Eickhoff S. B. Stephan K. E. Mohlberg H. Grefkes C. Fink G. R. Amunts K. (2005). A new SPM toolbox for combining probabilistic cytoarchitectonic maps and functional imaging data. Neuroimage 25, 1325–1335 10.1016/j.neuroimage.2004.12.034 15850749

[B11] Feige B. Scheffler K. Esposito F. Di Salle F. Hennig J. Seifritz E. (2005). Cortical and subcortical correlates of electroencephalographic alpha rhythm modulation. J. Neurophysiol. 93, 2864–2872 10.1152/jn.00721.2004 15601739

[B12] Friston K. J. Holmes A. P. Worsley K. J. Poline J. P. Frith C. D. Frackowiak R. S. J. (1994). Statistical parametric maps in functional imaging: a general linear approach. Hum. Brain Mapp. 2, 210 10.1002/hbm.460010306 24578041

[B13] Friston K. J. Williams S. Howard R. Frackowiak R. S. J. Turner R. (1996). Movement-related effects in fMRI time-series. Magn. Reson. Med. 35, 346–355 10.1002/mrm.1910350312 8699946

[B14] Gastaut H. (1952). Electrocorticographic study of the reactivity of rolandic rhythm. Rev. Neurol. 87, 176–182 13014777

[B15] Goense J. B. M. Logothetis N. K. (2008). Neurophysiology of the BOLD fMRI signal in awake monkeys. Curr. Biol. 18, 631–640 10.1016/j.cub.2008.03.054 18439825

[B16] He B. Liu Z. (2008). Multimodal functional neuroimaging: integrating functional MRI and EEG/MEG. IEEE Rev. Biomed. Eng. 1, 23–40 10.1109/RBME.2008.2008233 20634915 PMC2903760

[B17] Henson R. Friston K. J. (2007). Convolution models for fMRI, in Statistical Parametric Mapping: the Analysis of Functional Brain Images Anonymous (Amsterdam: Elsevier), 178–192

[B18] Hjorth B. (1975). An on-line transformation of EEG scalp potentials into orthogonal source derivations. Electroencephalogr. Clin. Neurophysiol. 39, 526–530 10.1016/0013-4694(75)90056-5 52448

[B19] Horovitz S. G. Fukunaga M. de Zwart J. A. van Gelderen P. Fulton S. C. Balkin T. J. (2008). Low frequency BOLD fluctuations during resting wakefulness and light sleep: a simultaneous EEG-fMRI study. Hum. Brain Mapp. 29, 671–682 10.1002/hbm.20428 17598166 PMC6871022

[B20] Josephs O. Turner R. Friston K. (1997). Event-related fMRI. Hum. Brain Mapp. 5, 243–248 10.1002/(SICI)1097-0193(1997)5:4<243::AID-HBM7>3.0.CO;2-3 20408223

[B21] Kilner J. M. Mattout J. Henson R. Friston K. J. (2005). Hemodynamic correlates of EEG: a heuristic. Neuroimage 28, 280–286 10.1016/j.neuroimage.2005.06.008 16023377

[B22] Laufs H. Kleinschmidt A. Beyerle A. Eger E. Salek-Haddadi A. Preibisch C. (2003). EEG-correlated fMRI of human alpha activity. Neuroimage 19, 1463–1476 10.1016/S1053-8119(03)00286-6 12948703

[B23] Liu Z. Ding L. He B. (2006). Integration of EEG/MEG with MRI and fMRI. IEEE Eng. Med. Biol. Mag. 25, 46–53 10.1109/MEMB.2006.1657787 16898658 PMC1815485

[B24] Liu A. K. Belliveau J. W. Dale A. M. (1998). Spatiotemporal imaging of human brain activity using functional MRI constrained magnetoencephalography data: Monte Carlo simulations. Proc. Natl. Acad. Sci. U.S.A. 95, 8945–8950 10.1073/pnas.95.15.8945 9671784 PMC21182

[B25] Logothetis N. K. Pauls J. Augath M. Trinath T. Oeltermann A. (2001). Neurophysiological investigation of the basis of the fMRI signal. Nature 412, 157 10.1038/35084005 11449264

[B26] MacKay W. A. Crammond D. J. (1987). Neuronal correlates in posterior parietal lobe of the expectation of events. Behav. Brain Res. 24, 167–179 10.1016/0166-4328(87)90055-6 3606800

[B27] Mantini D. Perrucci M. G. Del Gratta C. Romani G. L. Corbetta M. (2007). Electrophysiological signatures of resting state networks in the human brain. Proc. Natl. Acad. Sci. U.S.A. 104, 13170–13175 10.1073/pnas.0700668104 17670949 PMC1941820

[B28] Marsden J. Werhahn K. Ashby P. Rothwell J. Noachtar S. Brown P. (2000). Organization of cortical activities related to movement in humans. J. Neurosci. 20, 2307–2314 10704506 10.1523/JNEUROSCI.20-06-02307.2000 PMC6772500

[B29] Moosmann M. Ritter P. Krastel I. Brink A. Thees S. Blankenburg F. (2003). Correlates of alpha rhythm in functional magnetic resonance imaging and near infrared spectroscopy. Neuroimage 20, 145–158 10.1016/S1053-8119(03)00344-6 14527577

[B30] Niazy R. K. Beckmann C. F. Iannetti G. D. Brady J. M. Smith S. M. (2005). Removal of FMRI environment artifacts from EEG data using optimal basis sets. Neuroimage 28, 720–737 10.1016/j.neuroimage.2005.06.067 16150610

[B31] Ohara S. Mima T. Baba K. Ikeda A. Kunieda T. Matsumoto R. (2001). Increased synchronization of cortical oscillatory activities between human supplementary motor and primary sensorimotor areas during voluntary movements. J. Neurosci. 21, 9377–9386 11717371 10.1523/JNEUROSCI.21-23-09377.2001 PMC6763917

[B32] Papakostopoulos D. Crow H. J. Newton P. (1980). Spatiotemporal characteristics of intrinsic evoked and event-related potentials in the human cortex, in Rhythmic EEG Activities and Cortical Functioning, eds Pfurtscheller G. Lopes da Silva F. H. Petsche H. (Amsterdam: Elsevier), 179–200

[B33] Parkes L. M. Bastiaansen M. C. M. Norris D. G. (2006). Combining EEG and fMRI to investigate the post-movement beta rebound. Neuroimage 29, 685–696 10.1016/j.neuroimage.2005.08.018 16242346

[B34] Pfurtscheller G. (1981). Central beta rhythm during sensorimotor activities in man. Electroencephalogr. Clin. Neurophysiol. 51, 253–264 10.1016/0013-4694(81)90139-5 6163614

[B35] Pfurtscheller G. Lopes da Silva F. H. (1999). Event-related EEG/MEG synchronization and desynchronization: basic principles. Clin. Neurophysiol. 110, 1842–1857 10.1016/S1388-2457(99)00141-8 10576479

[B36] Popivanov D. Mineva A. Krekule I. (1999). EEG patterns in theta and gamma frequency range and their probable relation to human voluntary movement organization. Neurosci. Lett. 267, 5–8 10.1016/S0304-3940(99)00271-2 10400235

[B37] Ritter P. Moosmann M. Villringer A. (2009). Rolandic alpha and beta EEG rhythms' strengths are inversely related to fMRI-BOLD signal in primary somatosensory and motor cortex. Hum. Brain Mapp. 30, 1168–1187 10.1002/hbm.20585 18465747 PMC6870597

[B38] Rosa M. J. Kilner J. Blankenburg F. Josephs O. Penny W. (2010). Estimating the transfer function from neuronal activity to BOLD using simultaneous EEG-fMRI. Neuroimage 49, 1496–1509 10.1016/j.neuroimage.2009.09.011 19778619 PMC2793371

[B39] Rushworth M. F. S. Krams M. Passingham R. E. (2001). The attentional role of the left parietal cortex: the distinct lateralization and localization of motor attention in the human brain. J. Cogn. Neurosci. 13, 698–710 10.1162/089892901750363244 11506665

[B40] Rushworth M. F. S. Nixon P. D. Renowden S. Wade D. T. Passingham R. E. (1997). The left parietal cortex and motor attention. Neuropsychologia 35, 1261–1273 10.1016/S0028-3932(97)00050-X 9364496

[B41] Sadaghiani S. Scheeringa R. Lehongre K. Morillon B. Giraud A. L. D'Esposito M. (2012). Alpha-band phase synchrony is related to activity in the fronto-parietal adaptive control network. J. Neurosci. 32, 14305–14310 10.1523/JNEUROSCI.1358-12.2012 23055501 PMC4057938

[B42] Sadaghiani S. Scheeringa R. Lehongre K. Morillon B. Giraud A. L. Kleinschmidt A. (2010). Intrinsic connectivity networks, alpha oscillations, and tonic alertness: a simultaneous electroencephalography/functional magnetic resonance imaging study. J. Neurosci. 30, 10243–10250 10.1523/JNEUROSCI.1004-10.2010 20668207 PMC6633365

[B43] Salmelin R. Hámáaláinen M. Kajola M. Hari R. (1995). Functional segregation of movement-related rhythmic activity in the human brain. Neuroimage 2, 237–243 10.1006/nimg.1995.1031 9343608

[B44] Salmelin R. Hari R. (1994). Spatiotemporal characteristics of sensorimotor neuromagnetic rhythms related to thumb movement. Neuroscience 60, 537–550 10.1016/0306-4522(94)90263-1 8072694

[B45] Scheeringa R. Fries P. Petersson K. Oostenveld R. Grothe I. Norris D. G. (2011). Neuronal dynamics underlying high- and low-frequency EEG oscillations contribute independently to the human BOLD signal. Neuron 69, 572–583 10.1016/j.neuron.2010.11.044 21315266

[B46] Sclocco R. Tana M. G. Visani E. Gilioli I. Panzica F. Franceschetti S. (2012). EEG-informed fMRI analysis during a hand grip task, in Engineering in Medicine and Biology Society (EMBC), 2012 Annual International Conference of the IEEE (San Diego, CA), 4712–4715 10.1109/EMBC.2012.6347019 23366980

[B47] Talairach J. Tournoux P. (1988). Co-Planar Stereotaxic Atlas of the Human Brain. Stuttgart: Thieme Medical Publishers

[B48] Tallon-Baudry C. Bertrand O. (1999). Oscillatory gamma activity in humans and its role in object representation. Trends Cogn. Sci. 3, 151–162 10.1016/S1364-6613(99)01299-1 10322469

[B49] Turak B. Louvel J. Buser P. Lamarche M. (2001). Parieto-temporal rhythms in the 6–9 Hz band recorded in epileptic patients with depth electrodes in a self-paced movement protocol. Clin. Neurophysiol. 112, 2069–2074 10.1016/S1388-2457(01)00652-6 11682345

[B50] Visani E. Agazzi P. Canafoglia L. Panzica F. Ciano C. Scaioli V. (2006). Movement-related desynchronization-synchronization (ERD/ERS) in patients with Unverricht–Lundborg disease. Neuroimage 33, 161–168 10.1016/j.neuroimage.2006.06.036 16904345

[B51] Visani E. Minati L. Canafoglia L. Gilioli I. Granvillano A. Varotto G. (2011). Abnormal ERD/ERS but unaffected BOLD response in patients with Unverricht–Lundborg disease during index extension: a simultaneous EEG-fMRI study. Brain Topogr. 24, 65–77 10.1007/s10548-010-0167-5 21107673

[B52] Willemse R. B. de Munck J. C. Verbunt J. P. A. van't Ent D. Ris P. Baayen J. C. (2010). Topographical organization of mu and Beta band activity associated with hand and foot movements in patients with perirolandic lesions. Open Neuroimag. J. 4, 93–99 10.2174/1874440001004010093 21347203 PMC3043303

