# Supplementary material for: EEG-informed fMRI analysis during a hand grip task: estimating the relationship between EEG rhythms and the BOLD signal
Source: Front Hum Neurosci. 2014 Apr 1;8:186. doi: 10.3389/fnhum.2014.00186 (PMC3978331; doi:10.3389/fnhum.2014.00186)
Supplement: Supplementary file 1 [file Presentation1.PDF]

Supplementary Material

EEG-informed fMRI analysis during a hand grip task: estimating the relationship between EEG rhythms and the BOLD signal

Roberta Sclocco[1]*, Maria G. Tana[1,2,3], Elisa Visani[4], Isabella Gilioli[4], Ferruccio Panzica[4], Silvana Franceschetti[4], Sergio Cerutti[1], Anna M. Bianchi[1]

[1]Department of Electronics, Information and Bioengineering (DEIB), Politecnico di Milano, Milan, Italy
[2]BIND – Behavioral Imaging and Neural Dynamics Center, University "G. d'Annunzio", Chieti, Italy
[3]Department of Medicine and Aging Science, University "G. d'Annunzio", Chieti, Italy
[4]Fondazione IRCSS Istituto Neurologico "C. Besta", Milan, Italy

*** Correspondence:** Roberta Sclocco, Department of Electronics, Information and Bioengineering (DEIB), Politecnico di Milano, Via Golgi 39, Milan, 20133, Italy.
roberta.sclocco@polimi.it

Artifact correction on EEG data

In order to assess the good quality of artifact-corrected EEG data, we first examined the power spectra before and after gradient artifact removal. The comparison showed a reduction of gradient artifact peaks (artifact peaks are found on multiples of 10.5 Hz (TR/# slices, that is, 2s/21)) of five orders of magnitude (10^{-5}) after the removal procedure, demonstrating therefore the effectiveness of the chosen approach in removing gradient artifact (Figure SM1, top and middle panels).

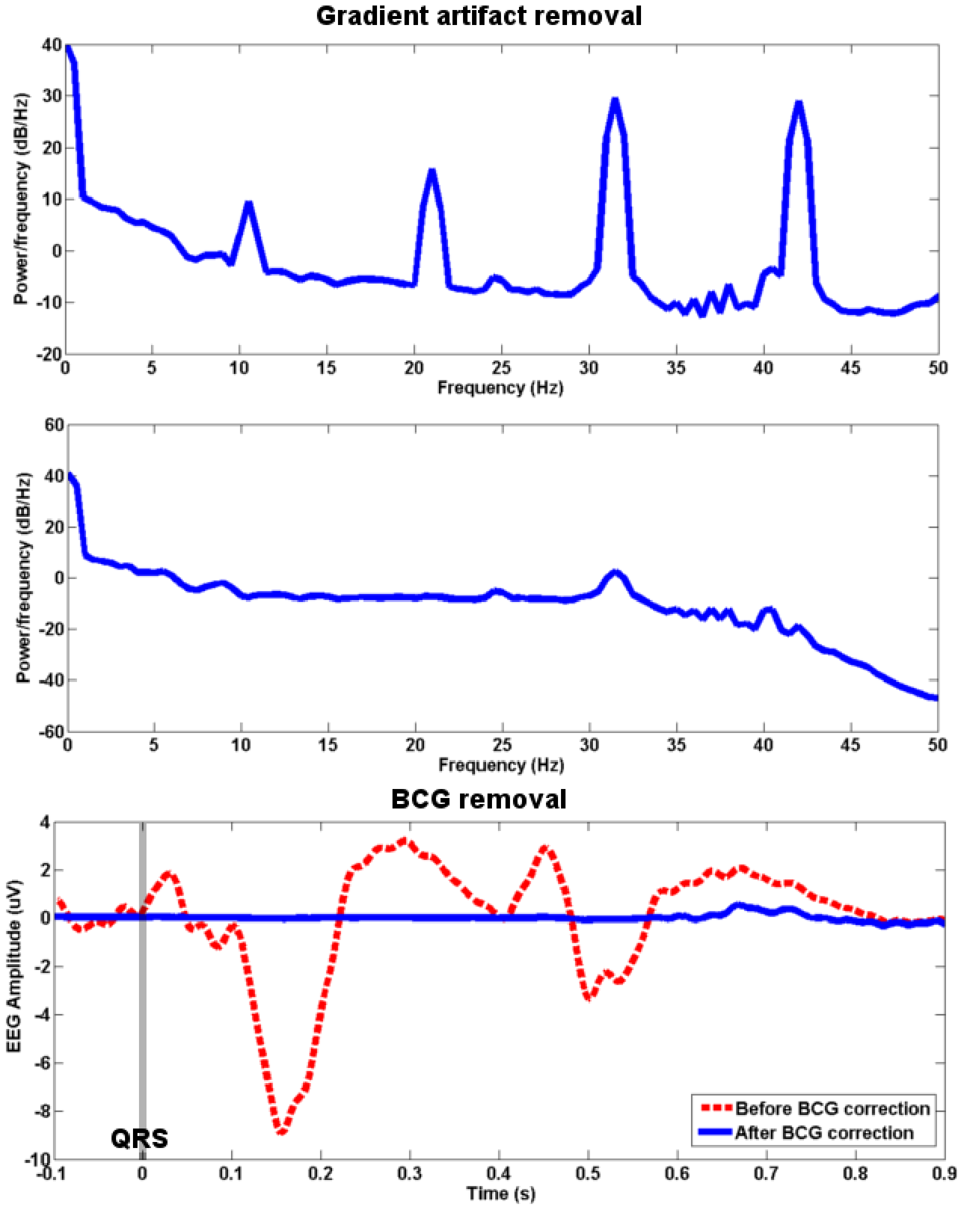

Figure SM1 Artifact removal on EEG signal for a representative subject Top panel: power spectrum of raw EEG data (C3 channel), with gradient-related peaks every 10.5 Hz. Middle panel: power spectrum of the same channel after removing gradient artifact. Bottom panel: averaging of 1 s epochs relative to each QRS onset (-100 ms – 900 ms) showing the effect of BCG artifact before correction (dotted red line) and the effectiveness of its removal (solid blue line).

We then checked the effectiveness of ballistocardiogram (BCG) removal by epoching data and examining them in the time domain. For each channel, a window of 1 second is taken every heartbeat, starting 100 ms before the QRS onset (Mantini et al. 2007a), and all the epochs are then averaged. The resulting curve obtained on data after applying optimal basis set (OBS) procedure shows a flat trend with respect to the one obtained from data before BCG removal (mean ± SD for C3 channel data is -0.0724 ± 2.9433 μV before BCG removal and 0.0432 ± 0.3456 μV after BCG removal), therefore demonstrating the effectiveness of the OBS approach in removing BCG artifact (Figure SM1, bottom panel). The OBS technique has indeed been proven to be particularly effective for the removal of the BCG artifact, which shows a considerable spatial variation across its occurences (Vanderperren et al. 2007): the method relies on the idea that principal component analysis (PCA) applied to all artifact occurrences in each channel separately makes possible to capture the temporal variations of the BCG artifact.

Furthermore, a recent study by (Vanderperren et al. 2010) comparing independent component analysis (ICA) and OBS approaches, showed that both the techniques yielded equally good results, and that OBS is more robust and easy to use since ICA methods required more parameter tuning.

References

Mantini, D., Perrucci, M.G., Cugini, S., Ferretti, A., Romani, G.L., and Del Gratta, C. (2007a). Complete artifact removal for EEG recorded during continuous fMRI using independent component analysis. *Neuroimage* 34, 598-607

Vanderperren, K., Ramautar, J., Novitski, N., De Vos, M., Mennes, M., Vanrumste, B., Stiers, P., et al. (2007). Ballistocardiogram artifacts in simultaneous EEG-fMRI acquisitions. *International Journal of Bioelectromagnetism* 9, 146-150

Vanderperren, K., De Vos, M., Ramautar, J.R., Novitskiy, N., Mennes, M., Assecondi, S., Vanrumste, B., et al. (2010). Removal of BCG artifacts from EEG recordings inside the MR scanner: A comparison of methodological and validation-related aspects. *Neuroimage* 50, 920-934.
